# Diversity and metabolic potentials of microbial communities associated with pollinator and cheater fig wasps in fig-fig wasp mutualism system

**DOI:** 10.3389/fmicb.2022.1009919

**Published:** 2022-11-18

**Authors:** Yiyi Dong, Zheng-Ren Zhang, Sandhya Mishra, Adam Chun-Nin Wong, Jian-Feng Huang, Bo Wang, Yan-Qiong Peng, Jie Gao

**Affiliations:** ^1^CAS Key Laboratory of Tropical Forest Ecology, Xishuangbanna Tropical Botanical Garden, Chinese Academy of Sciences, Menglun, China; ^2^School of Forest Resources and Conservation, University of Florida, Gainesville, FL, United States; ^3^University of Chinese Academy of Sciences, Beijing, China; ^4^Entomology and Nematology Department, University of Florida, Gainesville, FL, United States

**Keywords:** co-occurrence network, fig wasp, microbial community, whole-genome resequencing, interactions

## Abstract

Microbial symbionts can influence a myriad of insect behavioral and physiological traits. However, how microbial communities may shape or be shaped by insect interactions with plants and neighboring species remains underexplored. The fig-fig wasp mutualism system offers a unique model to study the roles of microbiome in the interactions between the plants and co-habiting insects because a confined fig environment is shared by two fig wasp species, the pollinator wasp (*Eupristina altissima* and *Eupristina verticillata*) and the cheater wasp (*Eupristina* sp1 and *Eupristina* sp2). Here, we performed whole genome resequencing (WGS) on 48 individual fig wasps (*Eupristina* spp.) from Yunnan, China, to reveal the phylogenetic relationship and genetic divergence between pollinator and congeneric cheater wasps associated with the *Ficus* trees. We then extracted metagenomic sequences to explore the compositions, network structures, and functional capabilities of microbial communities associated with these wasps. We found that the cheaters and pollinators from the same fig species are sister species, which are highly genetically divergent. Fig wasps harbor diverse but stable microbial communities. Fig species dominate over the fig wasp genotype in shaping the bacterial and fungal communities. Variation in microbial communities may be partially explained by the filtering effect from fig and phylogeny of fig wasps. It is worth noting that cheaters have similar microbial communities to their sister pollinators, which may allow cheaters to coexist and gain resources from the same fig species. In terms of metabolic capabilities, some bacteria such as *Desulfovibrio* and *Lachnospiraceae* are candidates involved in the nutritional uptake of fig wasps. Our results provide novel insights into how microbiome community and metabolic functions may couple with the fig-wasp mutualistic systems.

## Introduction

Microorganisms represent a predominant component of biodiversity and help maintain the function and stability of ecological systems (Weinbauer and Wenderoth, 2002; Maron et al., 2018; Escalas et al., 2019; Ratzke et al., 2020). Thus, exploring the composition and function of microbial communities in ecosystems contributes to understanding of ecological factors shaping biodiversity and species interactions (Zilber-Rosenberg and Rosenberg, 2008; Gibbons and Gilbert, 2015; Nazir et al., 2019), particularly those associated with herbivore insects and plants (Mithöfer and Boland, 2012; Casteel and Hansen, 2014; Zhu et al., 2014; Wielkopolan and Obrępalska-Stęplowska, 2016). For instance, microorganisms associated with herbivore insects can modify plant phytohormones and antiherbivore defenses (Casteel and Hansen, 2014). Microbial manipulation of plant chemistry and immunity could also affect plant susceptibility to herbivore insects (Lazebnik et al., 2014; Koduru, 2021). These examples demonstrate that the microorganisms can play critical roles in modulating plant-herbivore interactions.

In addition, insect-associated microorganisms could drive insect diversification and speciation (Vavre and Kremer, 2014). For instance, *Wolbachia* are widely associated with insects and can cause hybrid incompatibility in many species (Bordenstein et al., 2001; Serbus et al., 2008; Vavre and Kremer, 2014). Moreover, gut bacteria associated with *Drosophila* have been shown to affect the kin recognition of host insects and may change the reproductive investment of insects (Lizé et al., 2014). Co-speciation between host insects and their gut symbionts implies that parallel evolutionary processes might exist in the insects-bacteria system (Groussin et al., 2020). However, research has mainly focused on the crops and generalist insects.

Here, we explored the microbial diversity and potential metabolic functions in fig-wasp mutualism, which is a classic example of coevolution and obligatory pollinator system. Each fig species is pollinated by a specific pollinating fig wasp species (Janzen, 1979; Herre, 1989) and the fig wasps solely oviposit and develop within the figs (Ware and Compton, 1994). Generally, when the figs are in the receptive stage, the narrow ostiole is opened to allow the pollinator fig wasps access (Ware and Compton, 1994; Souza et al., 2015), and after their entry, the ostiole is closed (Nefdt and Compton, 1996). The pollinator fig wasps lay eggs or pollination inside of figs (mature offspring will fly away the maternal figs and enter a new round cycle), which is a relatively confined and stable environment for the development of fig wasps. The specific micro-habitat reduces the uncertain disturbances from surrounding environment and provides a unique model for studying the associated microorganisms and their potential roles in driving plant-insect interactions in obligatory mutualism system. However, due to the extremely tiny size of the fig wasps, so far, there was no further study on the specific regions of the microbe communities associated, and even fewer reports about the microbe community on the fig wasp. For a long time, the relationship between the fig and pollinator wasp was thought to be strictly species-specific. Namely, each specific fig wasp species provides one fig species with the pollination service, and in turn, the fig wasp completes its life cycle in the specific fig species (Wiebes, 1979; Rasplus, 1996). Moreover, cheater wasp species can also exist (Peng et al., 2008; Jandér and Herre, 2010; Zhang et al., 2019). The cheater species were found in two fig-wasp systems in Yunnan Province, China: *Eupristina altissima* (Hymenoptera: Chalcidoidea; pollinator) and *Eupristina* sp1 (Hymenoptera: Chalcidoidea; cheater) on *Ficus altissima* Blume (*Ficus*, Moraceae; Peng et al., 2008). *Eupristina verticillata* (pollinator) and *Eupristina* sp2 (cheater) on *Ficus microcarpa* Linnaeus (Martinson et al., 2014; Zhang et al., 2019, 2021). The pollinator and their pairs cheater are sister species on per fig species, and the coexistence between pollinator and cheater is common (Peng et al., 2008; Zhang et al., 2021). The diversity and functions of microorganisms associated with the different pollinator and wasp species are largely unknown.

In this study, we conducted illumina sequencing on various samples of fig-wasp systems to reveal the phylogenetic relationship and genetic divergence between pollinator and congeneric cheater wasps associated with the *Ficus* trees. We extracted microbial reads not mapped to the wasp genomes to explore (1) the diversity, compositions, and potential functions of microbial community associated with each fig wasp species; (2) microbial co-occurrence network patterns; (3) the assemblage patterns of microbial communities associated with pollinator and cheater fig wasps. The genomic data provides new insights on potential contributions of microbiome to fig-fig wasp mutualism and coevolution.

## Materials and methods

### Sample collection and genomic sequencing

**Figure 1:**
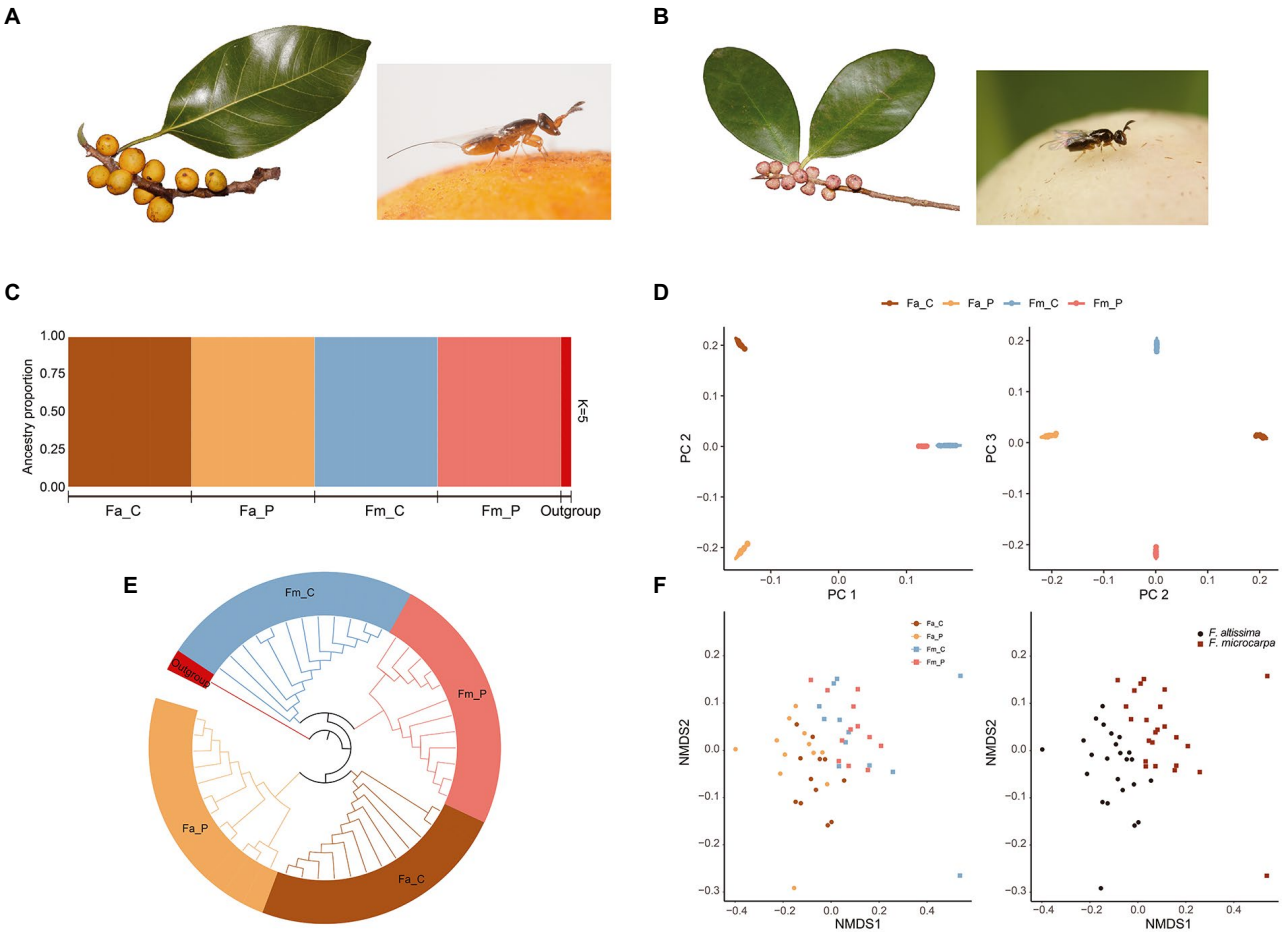
Phylogenetic, population genetic, and microbial similarity analysis of four fig wasp species. **(A)** *Ficus altissima* and its associated fig wasp; **(B)** *F. microcarpa* and its associated fig wasp. **(C)** *Faststructure* plot depicting ancestry proportions of each species in K = 5. **(D)** Genetic clusters identified based on the PC1 and PC2, PC2 and PC3 from the smartPCA analysis. **(E)** NJ tree constructed using the SNPhylo, the colors represented each species. **(F)** The Non-metric multidimensional scaling (NMDS) ordination analysis based on the composition of microbial communities. (Left) the Non-metric multidimensional scaling ordination (stress = 0.15, *p* < 0.01) produced with the Bray–Curtis dissimilarity distance metric. (Right) the same results of Left represent from different host figs. Fa_C represents the cheater of *F. altissima*; Fa_P represents the pollinator of *F. altissima*; Fm_C represents the cheater of *F. microcarpa*; Fm_P represents the pollinator of *F. microcarpa*.

From March to October 2020, figs (nearly phase that fig wasp offspring will emerge from figs) of *Ficus microcarpa* and *F. altissima*, were sampled at Xishuangbanna and nearby areas in Yunnan, China (Supplementary Table S1; Figures 1A,B). They mainly from three locations: Menglun town (20 individuals in total), Lushui (21 individuals in total), Chuxiong (7 individuals) with the distance of 500 and 280 kilometers. In order to get a well representative sampling of the fig wasps, for each location we sampled two crops, and we random picked figs covered several fig trees, we collected only one female pollinator or cheater wasp from each fig to ensure that each individual came from different foundress females. We cleaned the fig wasps with sterile water, and identified the pollinator and cheater species under a light microscope and stored them individually into tubes with 100% ethanol at −20°C. All the materials used for the sample collection were aseptically treated to avoid contamination. Genomic DNA was extracted from the whole body of each fig wasps using the QIAamp DNA Micro Kit (Qiagen, Hilden, Germany), and paired-end sequencing libraries with an insert size of 350 bp were constructed according to the Illumina library preparation protocol. Sequencing was carried out on the Illumina HiSeq 2,500 platform (Illumina Inc., San Diego, United States) with a target coverage of 20×. The sequencing data have been deposited in the NCBI, associated BioProject ID is PRJNA893401.

### Phylogenic tree and structure analysis of the fig wasp

The quality of raw sequence reads from fig wasps was assessed using FastQC.^1^ For raw sequencing reads, we used Trimmomatic (Bolger et al., 2014) to remove adapter sequences and cut off bases from either the start or the end of reads when the base quality was <20 We discarded the reads with fewer than 36 bases remaining after trimming. We then mapped all reads to the *Eupristina verticillata* reference genome (Zhang et al., 2020) with default parameters implemented in bwa-0.7.10 using the BWA-MEM algorithm (Li, 2013). Local realignment, and simultaneous SNP and indel discovery was performed using RealignerTargetCreator, IndelRealigner, and the HaplotypeCaller in GATK v3.2.2 (DePristo et al., 2011). We used MarkDuplicates in Picard^2^ to remove the potential PCR duplicates. After that, genotypes (in gVCF files) of all individuals were joined together by using the default hard filtering parameters as prescribed by GATK v3.2.2 best practices. Only sites with mapping quality ≥30 and base quality ≥30 were considered for calling variants in HaplotypeCaller (Supplementary Table S1). We used Vcftools (Danecek et al., 2011) to further filter low quality SNPs according to the following criteria: (1) only biallelic SNPs that were at least 5 bp away from any indel were retained; (2) genotypes with a genotype quality score (GQ) < 20, mapping quality score (MQ) < 40, and sites with sequencing depths (DP) < 10 were treated as missing; (3) SNPs with a genotype missing call rate ≥ 20% across the whole sample set were removed; (4) filtered the sites with minor alleles frequency (MAF) <0.05 sites, and removed the site where the proportion of heterozygous genotypes was >70% across the whole sample set. After these filtering steps, 397,502 SNPs were retained from all individuals for genetic analyses. To investigate the genetic structure of the fig wasps, we conducted three sets of analyses. (1) We ran *fastSTRUCTURE* (model-based clustering algorithm) with K = 1–20 and repeated the process 20 times with random seeds (Raj et al., 2014). Since linkage disequilibrium might violate the statistical assumptions involved in this test, we take one SNP from every 100 bp using the *- thin* in vcftools resulting in a dataset with 74,396 SNPs. (2) We assessed the distribution of genetic variance by principal component analysis (smartPCA) as implemented in EIGENSOFT version 6.0 (Patterson et al., 2006). (3) We constructed Neighbor-Joining (NJ) tree using SNPhylo (Lee et al., 2014), and with one individual from *Kradibia gibbosae* (pollinator of *F. tinctoria*) as the outgroup. Additionally, in order to check the genetic relationships of the fig wasps in this study with the samples in Hainan reported in previous study (Sun et al., 2011), we downloaded the 24 *COI* gene sequences of *Eupristina verticillata* from the paper Sun et al., 2011 (GenBankID:HM582244-HM582267) and chosen *E. koningsbergeri* (MK543378) and *E. altissima* (MK559358) as outgroups. We *de novo* assembled 23 mitochondrial genomes of *E. verticillata* using GetOrganelle (Jin et al., 2020), then extracted the *COI* gene in Geneious (Kearse et al., 2012), using BS-18 (HM582254) as the reference. We deposited the 23 COI genes in the National Center for Biotechnology Information GenBank under the accession number GenBank OP715733-OP715755. We reconstructed a maximum-likelihood (ML) phylogenetic tree based on the *COI* genes of a total of 47 *E. verticillata* and two outgroups (*E. koningsbergeri* and *E. altissima*). We used MAFFT (Katoh and Standley, 2013) to align 49 *COI* genes and ambiguously aligned fragments were removed by Gblocks (Talavera and Castresana, 2007). We used modelFinder (Kalyaanamoorthy et al., 2017) to select the best-fit nucleotide substitution model: TIM + F + I, then reconstructed ML tree by IQ-TREE (Nguyen et al., 2015). We inferred the support for the phylogenetic tree by bootstrapping with 10,000 ultrafast bootstraps (Minh et al., 2013).

### Bacterial and fungal community analysis

The analysis pipeline of the bacterial and fungal communities was composed of three steps. First, we used bwa v0.7.17^3^ to get the unmapped raw reads using the completely assembled reference genome of *E. verticillata* (Zhang et al., 2020). The unmapped reads were extracted from the mapping result files and estimated the quality reads *via* QualiMap v2.2.1 (Okonechnikov et al., 2016) and then individually paired-end assembled using Pandaseq (Masella et al., 2012). Finally, based on the blast v2.12.0^4^ with an expect value of <10 e^−10^, we aligned the unmapped reads to the SILVA database^5^ and Ribosomal Database Project^6^ to obtain the bacterial and fungal identifications. The blast outputs were performed analyses related to microbial diversity and function.

To estimate the similarity of microbial communities in the different samples, we conducted the non-parametric analysis of similarities analysis (ANOSIM), Non-metric multidimensional scaling (NMDS) with the Bray-Curtis distance and hierarchical clustering analysis with the ward.D2 algorithm (Murtagh and Legendre, 2014) on Euclidean distances. To characterize the corresponding relation in microbial communities and the phylogenetic structure of fig wasps, we visualized the topology structures of clusters of microbial communities and phylogenetic tree of fig wasps. Subsequently, we detected significance of the corresponding relation bycomparing to the null model (algorithm: quasiswap) based on 1,000 permutation samples in *PACo* package (Balbuena et al., 2013). Linear discriminant analysis effect size (LEfSe)^7^ was conducted to identify marker features that differentiate between from the pollinator and cheater fig wasp microbiomes, the different groups at the species level were determined using to explore whether the *Wolbachia* strains from the pollinator and cheater fig wasps show are genetically distinct, we conducted cluster analysis according to the similarity of *Wolbachia* compositions detected in the fig wasps associated with *F. microcarpa*.

To decipher the alpha-diversity and abundance of the fig wasp-associated bacterial and fungal communities, we calculated Shannon-Wiener and Chao 1 diversity indices of the fig wasp-associated microbial communities using the Vegan package (Oksanen et al., 2018). The compositions at the phylum and genus levels were visualized by stacked column charts. To evaluate the sampling effort, the microbial rarefaction and prediction curves were calculated by species richness in iNEXT (Hsieh et al., 2016) package.

### Bacterial and fungal co-occurrence patterns and interactions

To explore potential co-occurrence patterns and interactions in fig wasps-associated microbial communities, we performed network analysis using *igraph* package in the R programming environment (Csardi and Nepusz, 2006; R Development Core Team, 2018). Combining the fungal and bacterial communities from the same samples, we calculated the Spearman’s correlation coefficients and *p* value. Correlation coefficient lower than 0.8 and *p* value larger than 0.05 were filtered to generate a robust co-occurrence network. To describe the structure and parameters of the networks, we measured several network properties including connectance, average degree, diameter, modularity, and robustness. Connectance is the proportion of observed links among all possible interactions in the network, which descripts the complexity extent of network (Newman and Girvan, 2004; Landi et al., 2018). Average degree means the mean value of edges each node in the microbial network (Fornito et al., 2016). Diameter is defined as the largest length of the shortest paths between all pairs of vertices, and a measure of efficiency for the transmission resources across entire network (Luke, 2015). Modularity reflects the clustering extents to which compositions evaluates the clustering in microbial network (Newman et al., 2006). The modularity value ranges from −0.5 to 1, the value closer to 1 represents the network more clustering divided into density subgroups (Luke, 2015). Robustness evaluates the stability and resistance facing some species loss in the network (Dunne et al., 2002; Landi et al., 2018). Further, to evaluate the statistical significance of links in networks observed in samples, we compared the observed values to the null model generated by 500 times randomized network using the r2dtable algorithm (Dormann et al., 2009).

### Functional prediction of the fig wasp-associated bacterial and fungal communities

The functional annotation of fig wasp-associated fungi was performed using the FUNGuild (Nguyen et al., 2016) Guilds_v1.1.py script. To consider the confidence scores of predictions, we only included the “highly probable” and “probable” two identification levels in our function guilds. For the fig wasp-associated bacteria, we conducted the Tax4Fun2 to predict the bacterial metabolic functions (Wemheuer et al., 2020), and the FAPROTAX to detect the ecological function by collapse_table.py (Louca et al., 2016). The functional composition and enrichment were visualized with the bar plots and heatmaps.

## Results

### The phylogeny and genetic structure of the fig wasp species

The cross-validation procedure from the *fastSTRUCTURE* analysis identified K = 5 as the best parameter for the number of clusters in the samples. The five genetic clusters fit well with the species identification based on prior species category (Figure 1C). The four fig wasps were genetically independent and no genetic admix was observed between the pollinator and cheater wasps, and no further subgroups was appeared within each wasp species (Figure 1C) The genetic structure among the four fig wasp species was also supported by the Principal Component Analysis (PCA) and phylogenetic tree (Figures 1D,E). Together, the genetic analyses showed that individuals from each species showed a consistent genetic identity and the microbe compositions will not be affected by the genetic variation within species.

The ML tree based on *COI* gene of the pollinator and cheater wasps in this study showed that the pollinator wasps clustered with clade 1 (identified in Hainan islands), whereas the cheater wasps clustered with. Clade 2 (identified in Hainan; Supplementary Figure S1), and no wasp species in this study clustered with clade 3 (a unique cryptic species group in Hainan).

### The bacterial and fungal communities associated with the fig wasp species

**Figure 2:**
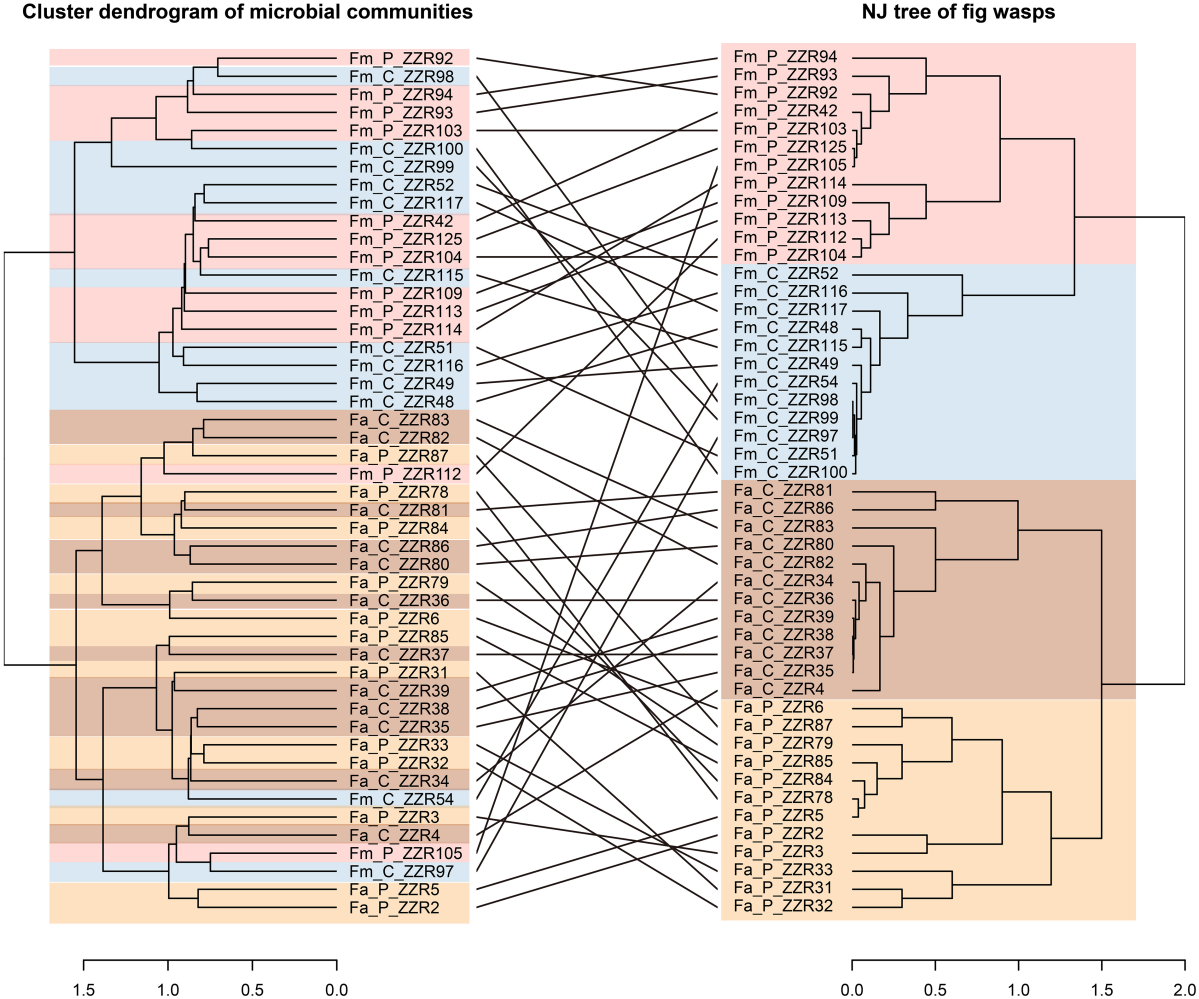
Left: the cluster dendrogram analysis based on the ward.D2 method using Euclidean distances of compositions of microbial communities in samples. The tip labels in the dendrogram are the sample codes. Right: the phylogenetic structure of fig wasps, (the corresponding relation: m^2^_XY_ = 0.72, *p* < 0.001, *n* = 1,000). Fa_C represents the cheater of *F. altissima*; Fa_P represents the pollinator of *F. altissima*; Fm_C represents the cheater of *F. microcarpa*; Fm_P represents the pollinator of *F. microcarpa*.

The NMDS plot and ANOSIM analysis reveal significant microbiome segregation among the different fig wasps, largely driven by the fig species (Figure 1F; stress = 0.15, *p* < 0.01) Microbial communities of *F. altissima-*associated wasps clustered separately from *F. microcarpa-*associated wasps. However, microbial communities are not significantly different between the cheater and pollinator wasps from the same host fig species (*F. altissima*: stress = 0.18, *p* = 0.06; *F. microcarpa*: stress = 0.11, *p* = 0.28). The same situation was also revealed by the co-phylogeny of the fig wasp and microbial communities. The NJ tree of the fig wasps revealed clearly species-specific clusters. However, the cluster dendrogram of microbial communities showed two clusters, the first one cluster consisting primarily of the microbial communities associated with the cheater and pollinator from *F. microcarpa*, and the second cluster consisting primarily of the microbial communities associated with the wasps from *F. altissima*, but admixed with two cheaters and pollinators from *F. microcarpa* (Figure 2). The certain congruence branches also were supported by the corresponding relation test, in which the microbial communities and phylogenetic topology have significant interaction intimacy (m^2^_XY_ = 0.72, *p* < 0.001, *n* = 1,000) than the null model.

The results from LEfSe analysis showed that Saccharomycetaceae, Cyanobacteria Chloroplast *Nicotiana tomentosiformis*, Cyanobacteria Chloroplast *Gossypium hirsutum*, and Cyanobacteria Chloroplast *Phaseolus acutifolius* were significantly more abundant in the pollinator wasps of *F. altissima* and we observed that 21 groups such as Proteobacteria, Enterobacteriales, Sphingobacteriales, *Vibrio*, and *Streptococcus* were significantly associated with the cheater of *F. altissima* (Supplementary Figure S2 A). Furthermore, 10 groups such as Burkholderiaceae, Rickettsiales Mitochondria *Hordeum*, Prevotellaceae, and *Wolbachia* were significantly more abundant in the cheater wasps of *F. microcarpa* (Supplementary Figure S2 B), and 35 groups were significantly abundant in the pollinator wasps of *F. microcarpa.*

Interestingly, *Wolbachia* was detected in *F. microcarpa*-associated fig wasps but not in *F. altissima*-associated fig wasps. Cluster analysis revealed two clusters, the first including 22.22 percent pollinators and 77.78 percent cheaters of *F. microcarpa* and the second containing 66.67 percent pollinators and 33.33 percent cheaters (Supplementary Figure S3).

### The bacteria and fungi diversity and composition analysis

**Figure 3:**
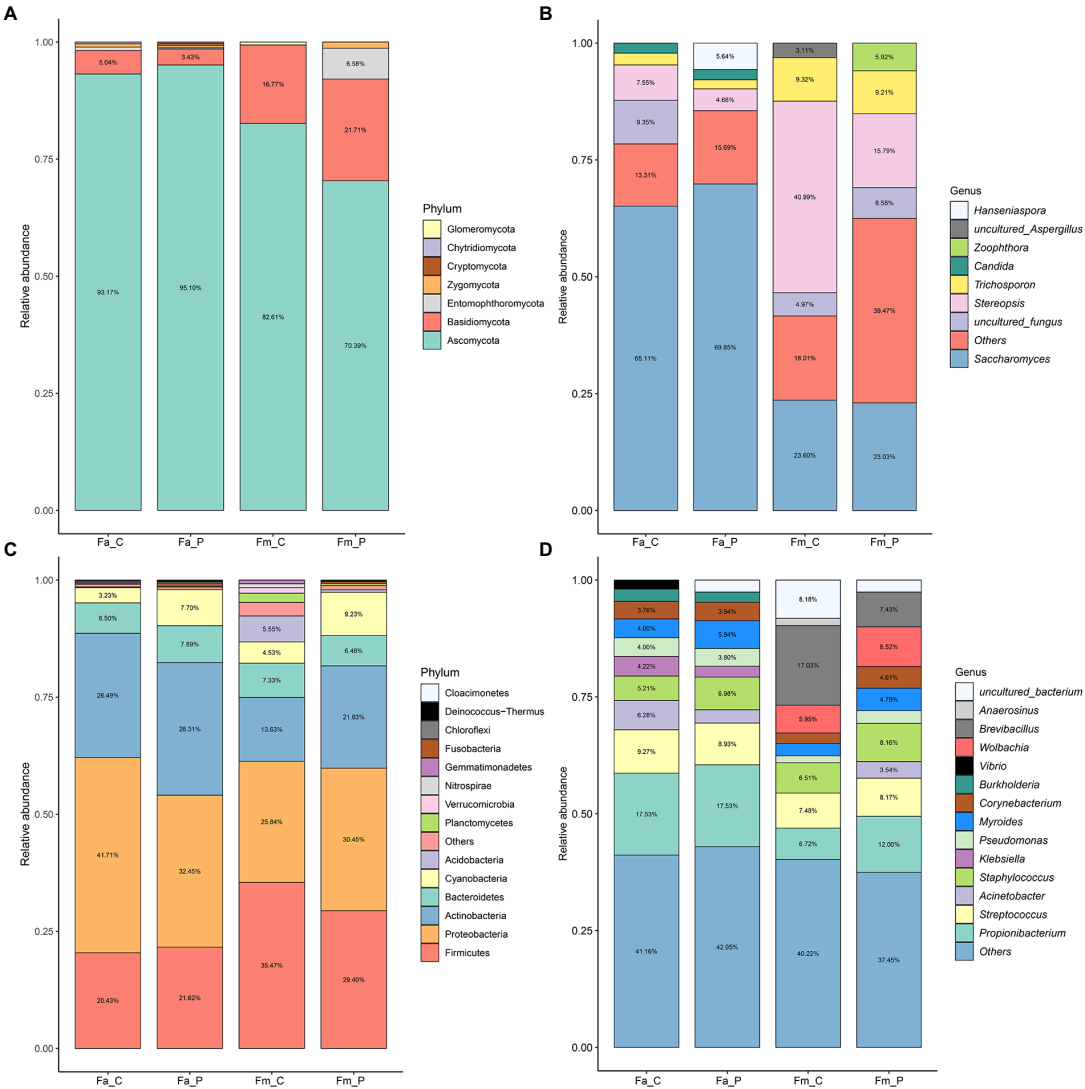
The relative abundance of fungal and bacterial communities in four different samples from fig wasps-associated microbial communities. **(A)** Fungal compositions at phylum level. **(B)** Fungal compositions at genus level. **(C)** Bacterial compositions at phylum level. **(D)** Bacterial compositions at genus level. The percentage is the relative abundance, the values less than 3% were not shown in panel. Fa_C represents the cheater of *F. altissima*; Fa_P represents the pollinator of *F. altissima*; Fm_C represents the cheater of *F. microcarpa*; Fm_P represents the pollinator of *F. microcarpa*.

In terms of fungal communities, alpha diversity indices were generally not different among the different fig wasps (Supplementary Figures S4 A,B). The Shannon diversity index (mean value) ranges from 1.12 (cheater of *F. altissima*) to 1.59 (pollinator of *F. microcarpa*), whereas the Chao 1 diversity index was not significant differences among samples. The bacterial alpha diversity also showed minimal differences among the fig wasp species (Supplementary Figures S4 C,D). The samples of *F. altissima* pollinator had a significant lower Chao 1 index than those samples from *F. microcarpa*. In terms of taxonomic composition, the fungal communities were dominated by 7 phyla: Ascomycota, Basidiomycota, Entomophthoromycota, Zygomycota, Cryptomycota, Chytridiomycota, and Glomeromycota (Figures 3A,B). The Ascomycota was the most abundant group (70.39–95.10%) followed by Basidiomycota (3.43–5.04%). The *Saccharomyces* was the dominant genus in the samples from cheater (65.11%), pollinator (69.85%) of *F. altissima*, and pollinator of *F. microcarpa* (23.03%). Whereas the *Stereopsis* genus was most prominent in the sample from cheater of *F. microcarpa* (40.99%).

More bacterial phyla were detected and the top 5 were Proteobacteria, Firmicutes, Actinobacteria, Chloroflexi, and Acidobacteria (Figures 3C,D). The relative abundance of Proteobacteria was higher in cheaters (41.71%), pollinators (32.45%) of *F. altissima*, and pollinators of *F. microcarpa* (30.45%), whereas the dominant phylum was Firmicutes in cheaters of *F. microcarpa* (35.47%). At bacterial genus level, except for cheaters of *F. microcarpa*, *Propionibacterium* was the most abundant genus in all other samples. *Wolbachia* was only observed in cheaters (5.95%) and pollinators (8.52%) of *F. microcarpa*.

Moreover, the individual microbial compositions at phylum level were visualized (Supplementary Figures S5 A,B), indicating microbial compositions among samples were generally consistent. The rarefaction curves indicated that samples in this study did not reach the asymptotic platform (Supplementary Figures S6 A,B).

### The bacterial and fungal community co-occurrence patterns and interactions

Across all networks from our samples that integrated the bacterial and fungal communities, the r2dtable algorithm showed that network links were non-random and mainly positive significant interactions (Supplementary Figure S7), which may reflect the microbial groups trend to cooperation with each other in microenvironment of fig. The network from the cheater wasps of *F. altissima* consisted of 228 nodes (genera), 1,333 positive edges and 1 negative edge. While the network in pollinator of *F. altissima* had 244 nodes and 1,006 positive interactions. The network from cheater of *F. microcarpa* was composed of 441 nodes, 6,649 positive links and 2 negative links, whereas 410 nodes and 2,745 positive links formed the network in pollinator from *F. microcarpa*. The co-occurrence network analysis showed that Ascomycota, Proteobacteria, and Firmicutes are the most abundant nodes, this is consistent with results from the relative abundance of microbial communities. For each co-occurrence network, the maximum connectance and robustness were 0.21 and 0.99, respectively (Table 1). Furthermore, the highest average degree was observed in microbial communities from cheater of *F. microcarpa* (35.10). Samples from cheater of *F. microcarpa* showed the lowest modularity (0.37) and the network diameters of samples from *F. altissima* were less than samples from *F. microcarpa*.

**Table 1:** The parameters statistics for the microbial co-occurrence networks.

	**Connectance**	**Average degree**	**Diameter**	**Modularity**	**Robustness**
Fa_C	0.22	11.70	3	0.87	0.99
Fa_P	0.21	10.16	4	0.86	0.98
Fm_C	0.21	35.10	11	0.37	0.99
Fm_P	0.21	15.96	10	0.88	0.99

Fa_C represents the cheater of *F. altissima*; Fa_P represents the pollinator of *F. altissima*; Fm_C represents the cheater of *F. microcarpa*; Fm_P represents the pollinator of *F. microcarpa*.

### The bacteria and fungi functional prediction

**Figure 4:**
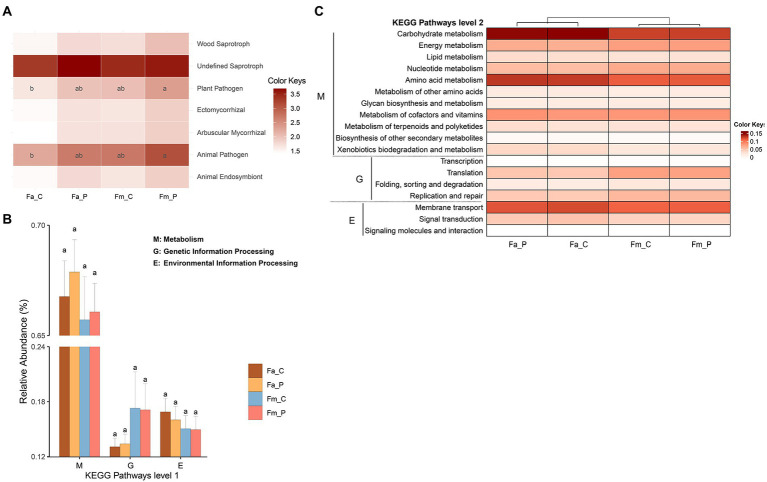
The function prediction of microbial communities. **(A)** Heatmap plot of fungal functional guilds produced by FUNGuild analysis. **(B)** Bar plots of the prediction Kyoto Encyclopedia of Genes and Genomes (KEGG) pathway level 1 obtained from Tax4Fun2 for each sample of bacterial community. **(C)** Heatmap of the functional abundances at the KEGG metabolic pathway level 2 obtained from Tax4Fun2 for each sample of bacterial community. The color bar reflects the value of functional abundance. The letters in each panel were calculated by the least significant difference (LSD) test, the same letter represents no differ significantly. The relative abundance of pathways with 0.05 cutoff (not shown). Fa_C represents the cheater of *F. altissima*; Fa_P represents the pollinator of *F. altissima*; Fm_C represents the cheater of *F. microcarpa*; Fm_P represents the pollinator of *F. microcarpa*.

Based on the fungal guild classification identified by the FUNGuild, 7 ecological functional guilds were detected (Figure 4A). The guilds were comprised of wood saprotroph, undefined saprotroph, plant pathogen, ectomycorrhizal, arbuscular mycorrhizal, animal pathogen, and animal endosymbiont. The most abundant guild was undefined saprotroph, followed by animal pathogen. The least significant difference (LSD) test showed that the animal pathogen and plant pathogen guilds were significantly more abundant in the samples from pollinator of *F. microcarpa* than in samples from cheater of *F. altissima.* The remaining ecological functional guilds showed non-significant difference among the samples.

All samples showed highly similar distribution patterns of the Kyoto Encyclopedia of Genes and Genomes (KEGG) pathways level 1 and 2 (Figures 4B,C). The bacterial communities in samples revealed that the predicted KEGG pathway level 1 includes metabolism, genetic information processing, environmental information processing, cellular processes, organismal systems, and human diseases. We set a cutoff of relative abundance > 0.05 to present the pathways in the results. At the KEGG metabolic pathway level 2, the pathways related to the metabolism (such as carbohydrate metabolism, energy metabolism and amino acid metabolism), genetic information processing, and environmental information processing were highly represented. No significant difference in KEGG pathways was detected among the different wasp bacterial communities. The ecological functions of bacteria *via* the FAPROTAX dataset showed that 74 functional groups were observed across the samples, with chemoheterotrophy, animal parasites or symbionts, aerobic chemoheterotrophy, and fermentation being the most abundant functional groups (Supplementary Figure S8).

## Discussion

### The cheater and pollinator fig wasps are highly divergent genetic lineages

In fig-fig wasp systems, coexisting cheaters and pollinators have similar morphological features but different pollination behavior. Whether these cheaters are phylogenetically unrelated from the pollinator or they evolved from the pollinator lineage is an controversial topic of research for biologists (Kong, 2014; Zhang et al., 2019). The first cheater case (*Ceratosolen galili*) found in *Ficus sycomorus* from Africa was due to the host plant shifting of fig wasps (Compton et al., 1991). Zhang et al. (2021) suggested that the cheaters and pollinators of *F. micorcarpa and F. altissima* were two distinct events but the cheater and pollinator from each host fig were closely related species, based on their mitochondria *COI* gene. However, this result was based on single gene evidence. In this study, the whole genome-wide sequence provides strong support that the cheaters and pollinators from the same fig species are sister species and are highly divergent. In nature, coexistence of cheaters and pollinators is also found in the yucca and yucca moth system (Pellmyr et al., 1996; Pellmyr and Leebens-Mack, 1999), where extensive hybridization serves as a general mechanism limiting the cheaters’ expansion (Segraves et al., 2005). However, no admixture between the cheaters and pollinators was found in our study, suggesting that they are completely reproductive-isolated. Our samples showed significant interspecific divergence but strong intraspecific genetic consistency in Xishuangbanna and the neighboring area.

### The microorganisms associated with fig wasps are extremely diverse

The common bacterial phyla associated with insects are reported to be Protobacteria, Firmicutes, Bacteroidetes, and Actinobacteria (Warnecke et al., 2007; Colman et al., 2012; Jones et al., 2013; Receveur et al., 2020). Ascomycota is a particularly predominant phylum in fungal communities (Douglas, 2015; Sharma et al., 2018; Morales-Rodriguez et al., 2019). Our results also support the previous findings with common microorganisms associated with other insects, and all the common phyla mentioned above are detected in this study.

Moreover, our results revealed more diverse and complex composition of fig wasp-associated microbial communities than documented. A previous report based on unmapped raw genomic sequence data of *Ceratosolen solmsi* (pollinator of *Ficus hispida*) identified 158 genera of fungi belonging to two phyla: Ascomycota (92.3%) and Basidiomycota (7.7%; Niu et al., 2015a). Except for the consistent with the results on dominant two phyla, we detected extra five fungal phyla more than 200 genera in fungal communities associated with our fig wasp specimens. Furthermore, compared to the results from Niu et al. (2015b), we documented greater variation in bacterial composition. They found the bacterial communities consisted of 43 genera belonging to 6 phyla from four fig wasp species (*Ceratosolen solmsi* Mayr, *Apocrypta bakeri* Joseph, *Philotrypesis pilosa* Mayr, and *Philotrypesis* sp. Forster), which differ in their phylogenetic relationships and diets but coexist in *Ficus hispida*. Among them, the pollinator wasp *C. solmsi* is dominated by Protobacteria (65.3%) and Actinobacteria (23.9%) in the associated bacterial community, using the Amplified ribosomal DNA restriction analysis (ARDRA) method (Niu et al., 2015b). A total of more than 400 genera, representing 19 different bacterial phyla were collected across our fig wasp samples, whereas Protobacteria, Firmicutes, and Actinobacteria were the most abundant bacterial groups in different samples. Overall, we observed more microbial groups which have not been identified in previous reports on fig wasps, such as the Entomophthoromycota, Zygomycota, Chloroflexi, Cyanobacteria, and Nitrospirae.

We argue that these differences in microbial composition could be attributed to several reasons. Previous studies showed that ARDRA has a much lower resolution than high throughput sequencing that is more sensitive and effective and may fail to capture diverse and abundant microbial taxa (Smit et al., 1997; De Mandal et al., 2015; Bailón-Salas et al., 2017; Hu et al., 2021). The different sexes of wasps used in the different studies may also contribute to the different results. Niu et al. (2015a) only detected Ascomycota and Basidiomycota strains in fungi groups associated with male individuals, whereas females were sampled in this study. The female and male are dimorphic in morphology and behavior in fig wasps, generally, wingless male mate within their natal fig fruits and never leave, whereas winged males disperse to mate (Weiblen, 2002). However, females must fly away natal fig to search proper fig to start a new life cycle, which means high movement ability may provide females more chance to contact extra microbial groups.

### Similarity in compositions and steady network structures of microbial communities between pollinator and cheater wasps

Several studies have suggested that host genotype has a bigger influence than environment in shaping microbial communities (Brucker and Bordenstein, 2012; Sanders et al., 2014). In this study, we observed that microbial taxa significantly varied among samples from two different *Ficus* tree species. As an obligate mutualism system, the figs are associated with their specific pollinator and non-pollinating wasps (Cook and Segar, 2010), whereas only few non-pollinating wasp species can enter the syconium for oviposition (West et al., 1996; Peng et al., 2005). The host fig might influence the microbial community of the pollinator wasps within the fig syconium. In addition, even in the same syconia, the bacterial community was also affected by the phylogeny and diet structure of host wasp (Sun et al., 2011). The fig wasps in this study are all phytophagous, and both pollinator wasps *Eupristina verticillata* (host fig: *F. microcapa*) and *Eupristina altissima* (host fig: *F. altissima*) belong to the genus *Eupristina* (Agaonidae). They have been separated for at least 28.83 Mya (Cruaud et al., 2022), indicating bacterial communities might have introduced and diverged through the long evolutionary time scale. Thus, we indicate that the microbial communities of fig wasps were influenced by the environment (syconia of different fig species) and the phylogenetic relationship of the host fig wasps themselves.

However, fig species has more dominating influence than fig wasp genotype in shaping the microbial communities. The correlations with the cluster dendrograms of microbial community compositions and the NJ tree dendrograms of fig wasps further confirmed this idea. Although a few of Fm samples are clustered into the Fa cluster (Figure 2), this suggests that the microbial compositions associated with fig wasps may (not completely accurate) represent the evolutionary relationship of fig wasps and also be impacted by the figs. The microbial communities showed more similarities between the pollinator and cheater wasps from the same fig species, which can be attributed to three potential reasons: First, the figs play filtering roles in avoiding external disturbances. The pollinator and their pair cheater can be observed in one fig, which means they can have contact with one other, leading to the horizontal transmission of the microorganisms, though coexistence in the same syconia is considered rare (~9%; Peng et al., 2008; Zhang et al., 2019). Second, the divergence time between the cheater and pollinator was 3.13 Mya in the *F. altissima* and 0.91 Mya in the *F. microcarpa*, both were significantly shorter than the separation time between wasps associated with different host fig species (28.83 Mya; Jie Gao et al., unpublished work). The third possible factor is that the cheater may mimic the pollinator’s microbial composition, which helps cheater to hide against recognition of figs. Figs have the ability to abort syconia with too many non-pollinator wasp offspring, thereby sanctioning cheaters and ensuring better pollination services from pollinators. This means the host figs trend to allocate more resource to beneficial pollinators (Jandér and Herre, 2016). In turn, this similarity in microbial community may be critical to cheaters to gain more resources from figs. Research in Xishuangbanna demonstrated that local *F. microcarpa* lack the host sanctions (Zhang et al., 2021). We speculated that the microbial communities of pollinator wasps might interact with the fig to affect the chemical signals of figs and inhibit of host sanction because the fig wasps rely on volatile compounds for locating the host figs (Ware et al., 1993) Bacteria associated with oviposition resources have been shown to influence the oviposition preference of flies (Zheng et al., 2013). Furthermore, the fungal compositions are markedly diverse at different developmental stages of figs, especially, in the stages during and after entering pollinators, indicating that fungi may provide volatile cues to fig wasps to track figs (Martinson et al., 2012). The exact role of microorganisms in mediating the fig’s recognition and oviposition behaviors of fig wasp merits further investigation.

The low to nonexistent host sanctions on the non-pollinator wasps promote the evolution of cheaters, causing the wasp undergo considerable morphological and genomic evolution to adapt to the extreme environment in the closed syconium (Zhang et al., 2021). We observed that the cheater and pollinators both have a stable network structure of microbial community with non-random network and high positive relationships. This is interesting because the prevalence of competition interaction is typically higher than cooperative in microbial communities (Palmer and Foster, 2022). The positive interactions may hold the key for stable mutualism between the fig, fig wasp., and microbial communities (Barberán et al., 2012; Chow et al., 2014; Gould et al., 2018). However, we also found that the network parameters of microbial communities from *F. altissima* are not exactly the same for the parameters from *F. microcarpa*. For instance, the high average degree and low modularity were observed in microbial communities of cheaters from *F. microcarpa*, which represents that each node has more links with other nodes but communities with low clustering density. We argued that this situation is due to the low effective nodes, which was supported by visualization (Supplementary Figure S7). Moreover, lower node number also results in higher diameter value (Scardoni and Laudanna, 2012), and the high network diameter presents lower efficiency for the transmission information and resources across entire microbial community in *F. microcarpa*. It is consistent with the smaller fig size in *F. microcarpa* than in *F. altissima* (observation results), implying fig wasps (such as cheaters and pollinators) have less coexistence chance in former than in latter.

### The microorganisms may couple with in maintaining nutrition and health of fig wasps

Microorganisms associated with insects can have major roles in insect nutrition and metabolism (Dillon and Dillon, 2004; Douglas, 2018; Jang and Kikuchi, 2020). The main functions of bacteria predicted in fig wasps include metabolism, fermentation and chemoheterotrophy, while the fungal guilds comprised of both pathogen and endosymbiont. Since the fig wasps lay eggs in the figs and incubate until they emerge from the gall flowers to mate, they can only obtain limited food from the figs, especially after mating (Janzen, 1979). Some groups of Proteobacteria, Firmicutes, Acidobacteria, and Bacteroidetes might contribute to nutrient provisioning to fig wasps, and may be involved in metabolic processes including nitrogen recycling and fixation (Hongoh, 2010; Desai and Brune, 2012; Köhler et al., 2012). The genus *Desulfovibrio* detected in our study could reduce sulfate (Kuhnigk et al., 1996), (Arias-Cordero et al., 2012) and participates in metabolic processes involving sulfate and acetate (Kuhnigk et al., 1996). Notably, some bacteria from genus *Enterobacter* are capable of degrade cell walls in the host plant (Xia et al., 2017), which might enhance utilization efficiency of fig resources for the fig wasps. The *Lachnospiraceae* that were identified at low abundance in our samples have high proteolytic and cellulolytic activities. They are considered an ecologically important constituent of the bacterial population under certain dietary conditions (Cotta and Forster, 2006). Another study in broad-headed bug demonstrated that infection of genus *Burkholderia,* common symbiotic bacteria, enhances insecticide resistance and fitness of host bug (Kikuchi et al., 2007, 2012).

Moreover, *Klebsiella*, *Enterocuccus*, and *Serratia* are capable of producing aggregation pheromone, a type of chemical compound that has been shown to cause locust swarming (Dillon et al., 2002). Fungi associated with the bark beetles can also synthesize specific aggregation pheromone 2-Methyl-3-buten-2-ol (Zhao et al., 2015). Other genera, including *Pseudomonas*, *Staphylococcus*, *Acinetobacter,* and *Pantoea,* contain numerous pathogenic species and can attract predators *via* chemical signaling (Chansang et al., 2010; Engel and Moran, 2013; Khan et al., 2014). This may account for the existence of diverse non-pollinator fig wasps, which some non-pollinator fig wasps tend to oviposit in figs that have been visited by pollinator fig wasps (Peng et al., 2005). Given that the microbial community composition and functions in the fig wasp system is severely underexplored, our study provides preliminary evidence on microbial community functions and their potential contributions to fig wasp physiology and health for further functional validation.

### The potential effects of *Wolbachia* on host fig wasp divergence and speciation

*Wolbachia* are the most common intracellular bacteria in arthropods and nematodes, and can affect host speciation through cytoplasmic incompatibility (Walker et al., 2021), sex pheromones, and mating behavior (Sharon et al., 2010). The incidence of *Wolbachia* infection is found to be pervasive in fig wasp (Dewayne Shoemaker et al., 2002; Haine and Cook, 2005; Ahmed et al., 2013). Earlier studies on a large-scale survey of the *Wolbachia* and their host fig wasps have pointed out that convergent incidence of *Wolbachia* is found in different fig wasp species, and suggested neither phylogeny nor ecological association among host species is consistent to the phylogenetic affinities of the *Wolbachia* infection (Dewayne Shoemaker et al., 2002; Haine and Cook, 2005). The *Wolbachia* cluster dendrogram in our study did not support absolute isolation between the cheater and pollinator wasps. However, a strong phylogenetic correlation between the pollinators and *Wolbachia* strains have been reported on the *F. microcapa* of Hainan islands (Sun et al., 2011). Their study showed that three clades (cryptic species, but the authors did not consider the existence of cheaters in *F. microcarpa*) of the pollinator wasps corresponded to three *Wobachia* strains. We further extracted the *COI* gene in our fig wasps and constructed the phylogenetic relationship with the three clades samples from Sun et al., 2011. The results showed that clade 1 was clustered with the pollinator wasps, and clade 2 corresponded to the cheater wasps (Supplementary Figure S1), indicating that the clade2 are very likely the cheaters of *F. microcarpa.*. Therefore, the effect of *Wolbachia* on speciation of fig wasps from *F. microcarpa* should be furtherly tested. Interestingly, the presence of *Wolbachia* infection was not observed in cheaters and pollinators from *F. altissima.* Although the reason of absence of *Wolbachia* infection is uncertain, the sampling individuals without *Wolbachia* infection, and lacking specific amplification primers of *Wolbachia* may account for failing to detect *Wolbachia* sequences from unmapped data.

## Conclusion

In the obligate pollinating systems, the cheater visitors are rare. However, the role of microorganism in this system still remains unknown. We analyzed the phylogenetic relationship between cheaters and pollinators, then explored diversity and contribution of microorganisms associated with cheater and pollinator wasps. Our study showed that the cheater and pollinator wasps are highly divergent genetic lineages. And their association microbial groups are extremely diverse, which may play essential roles in maintaining nutrient, health, and speciation of fig wasps. Besides, fig species dominate over the fig wasp genotype in shaping the microbial communities, which may help cheater to coexist with pollinators in figs.

## Data availability statement

The data presented in the study are deposited in the NCBI repository, accession number GenBank OP715733-OP715755. And the WGS data associated Bioproject number is PRJNA893401.

## Author contributions

JG, YD, and Y-QP conceived the study. JG, YD, and J-FH collected the samples. Z-RZ analyzed the genetic part data. YD analyzed the microbiome data. YD and JG wrote the manuscript. J-FH, Y-QP, SM, AW, and BW gave suggestions during the manuscript writing. All authors contributed to the article and approved the submitted version.

## Funding

This study was supported by grants from West Light Foundation of the Chinese Academy of Sciences and the National Natural Science Foundation of China (NSFC 32271591 and 32070487).

## Conflict of interest

The authors declare that the research was conducted in the absence of any commercial or financial relationships that could be construed as a potential conflict of interest.

## Publisher’s note

All claims expressed in this article are solely those of the authors and do not necessarily represent those of their affiliated organizations, or those of the publisher, the editors and the reviewers. Any product that may be evaluated in this article, or claim that may be made by its manufacturer, is not guaranteed or endorsed by the publisher.

## Footnotes

^1^ http://www.bioinformatics.babraham.ac.uk/projects/fastqc/

^2^ http://picard.sourceforge.net

^3^ https://github.com/sghignone/bwa

^4^ http://blast.ncbi.nlm.nih.gov/Blast.cgi?PAGE_TYPE=BlastDocs

^5^ https://www.arb-silva.de/

^6^ http://rdp.cme.msu.edu/index.jsp

^7^ http://huttenhower.sph.harvard.edu/galaxy/

## Supplementary material

SUPPLEMENTARY FIGURE S1 The ML tree of the pollinator and cheater wasps in this study (the Electric blue background) with the *Eupristina species* found in *F. microcarpa* in Hainan islands based on *COI* gene.

SUPPLEMENTARY FIGURE S2 The features of microbial communities. **(A)** LEfSe plot of biomarkers in communities from *F. altissima.* **(B)** LEfSe plot of biomarkers in communities from *F. microcarpa*. The threshold of discriminant score was 2.0. Fa_C represents the cheater of *F. altissima*; Fa_P represents the pollinator of *F. altissima*; Fm_C represents the cheater of *F. microcarpa*; Fm_P represents the pollinator of *F. microcarpa*.

SUPPLEMENTARY FIGURE S3 The cluster dendrogram produced by the ward.D2 method using Euclidean distances based on the compositions of *Wolbachia* bacteria in samples. The tip labels in the dendrogram are the sample codes; Fm_C represents the cheater of *F. microcarpa*; Fm_P represents the pollinator of *F. microcarpa*.

SUPPLEMENTARY FIGURE S4 The diversity indices patterns of fig wasps-associated fungi and bacteria. **(A,B)** The Shannon and Chao 1 diversity index of fungal communities. **(C,D)** The Shannon and Chao 1 diversity index of bacterial communities. The letters at the top of each panel were calculated by the least significant difference (LSD) test, the same letter represents no significant difference. Fa_C represents the cheater of *F. altissima*; Fa_P represents the pollinator of *F. altissima*; Fm_C represents the cheater of *F. microcarpa*; Fm_P represents the pollinator of *F. microcarpa*.

SUPPLEMENTARY FIGURE S5 The relative abundance of fungal and bacterial communities at phylum level in different individual samples. **(A)** Fungal compositions at phylum level. **(B)** Bacterial compositions at phylum level. Fa_C represents the cheater of *F. altissima*; Fa_P represents the pollinator of *F. altissima*; Fm_C represents the cheater of *F. microcarpa*; Fm_P represents the pollinator of *F. microcarpa*.

SUPPLEMENTARY FIGURE S6 The rarefaction curves of fungal community **(A)** and bacterial **(B)** among four different samples. Fa_C represents the cheater of *F. altissima*; Fa_P represents the pollinator of *F. altissima*; Fm_C represents the cheater of *F. microcarpa*; Fm_P represents the pollinator of *F. microcarpa*.

SUPPLEMENTARY FIGURE S7 The microbial co-occurrence networks based on filtered correlation coefficient (Spearman’s r > 0.8 and *p* value <0.05). **(A)** The co-occurrence network from the cheater of *F. altissima*. **(B)** The co-occurrence network from the pollinator of *F. altissima*. **(C)** The co-occurrence network from the cheater of *F. microcarpa*. **(D)** The co-occurrence network from the pollinator of *F. microcarpa*. The nodes in the co-occurrence network are the microbial genera, the color of nodes represents the phylum that the genus belongs to, the links in the network are the significant correlation in two nodes, the width of link line reflects the absolute value of correlation coefficient and the color of link line represents the positive (gray) and negative (red) correlation coefficient. Fa_C represents the cheater of *F. altissima*; Fa_P represents the pollinator of *F. altissima*; Fm_C represents the cheater of *F. microcarpa*; Fm_P represents the pollinator of *F. microcarpa*.

SUPPLEMENTARY FIGURE S8 Heatmap of the functional groups obtained from FAPROTAX for each sample of bacterial community. The color bar reflects the value of functional abundance. Fa_C represents the cheater of *F. altissima*; Fa_P represents the pollinator of *F. altissima*; Fm_C represents the cheater of *F. microcarpa*; Fm_P represents the pollinator of *F. microcarpa*.
