# Supplementary figures and images for: Diversity and metabolic potentials of microbial communities associated with pollinator and cheater fig wasps in fig-fig wasp mutualism system
Source: Front Microbiol. 2022 Nov 18;13:1009919. doi: 10.3389/fmicb.2022.1009919 (PMC9715610; doi:10.3389/fmicb.2022.1009919)

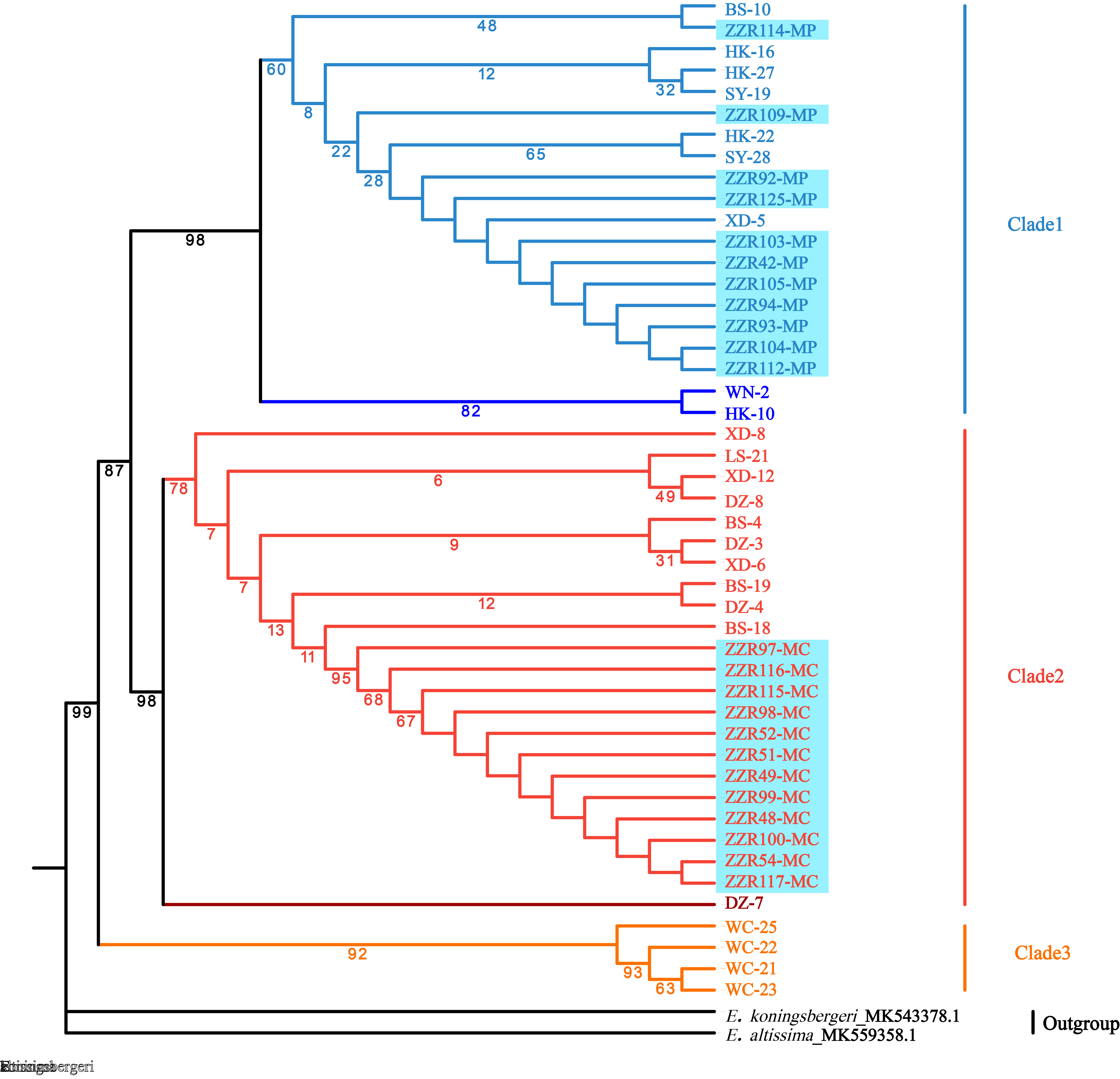

Supplement: SUPPLEMENTARY FIGURE S1 — The ML tree of the pollinator and cheater wasps in this study (the Electric blue background) with the Eupristina species found in F. microcarpa in Hainan islands based on COI gene. [file Image_1.JPEG]

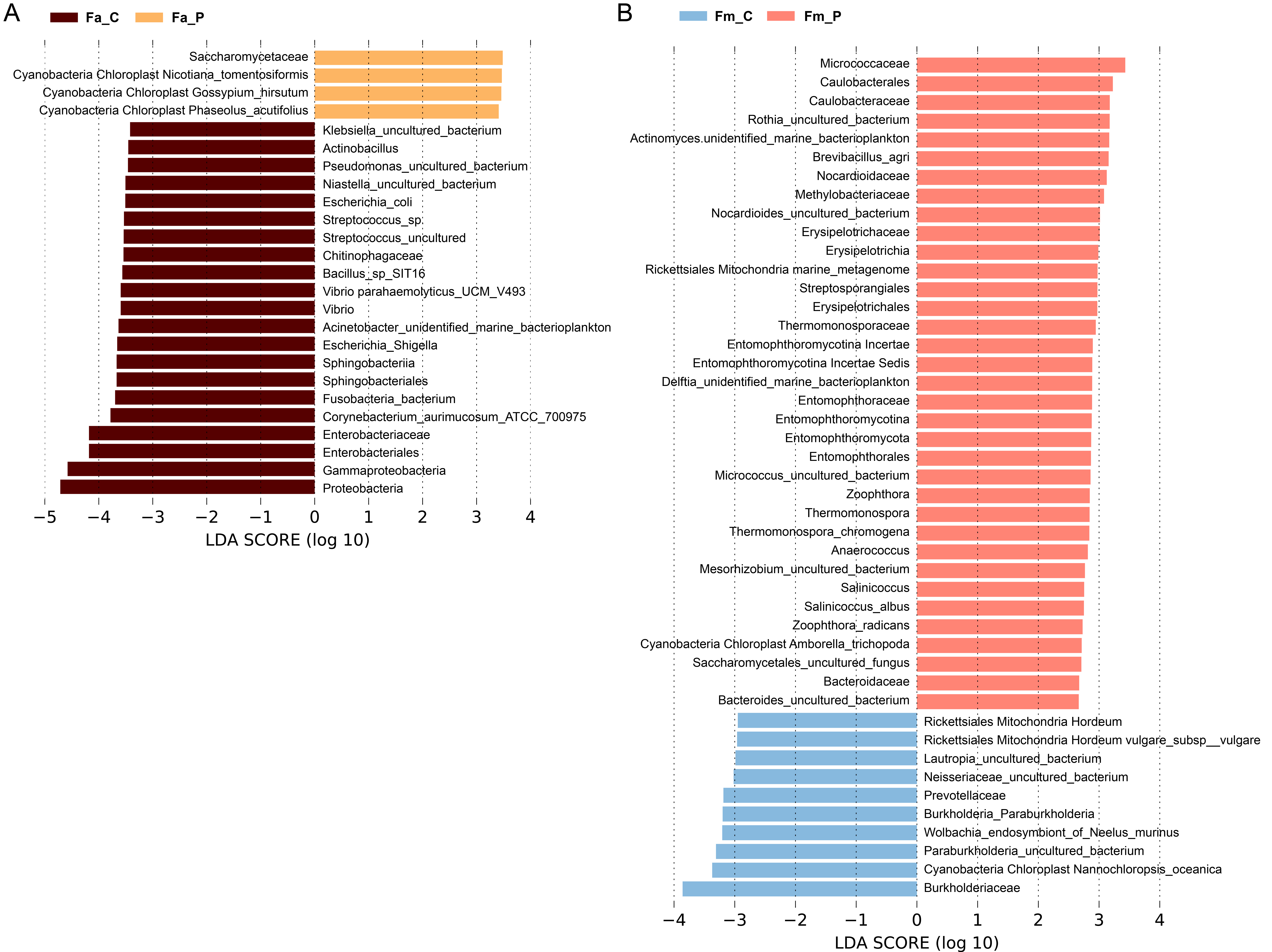

Supplement: SUPPLEMENTARY FIGURE S2 — The features of microbial communities. (A) LEfSe plot of biomarkers in communities from F. altissima. (B) LEfSe plot of biomarkers in communities from F. microcarpa. The threshold of discriminant score was 2.0. Fa_C represents the cheater of F. altissima; Fa_P represents the pollinator of F. altissima; Fm_C represents the cheater of F. microcarpa; Fm_P represents the pollinator of F. microcarpa. [file Image_2.JPEG]

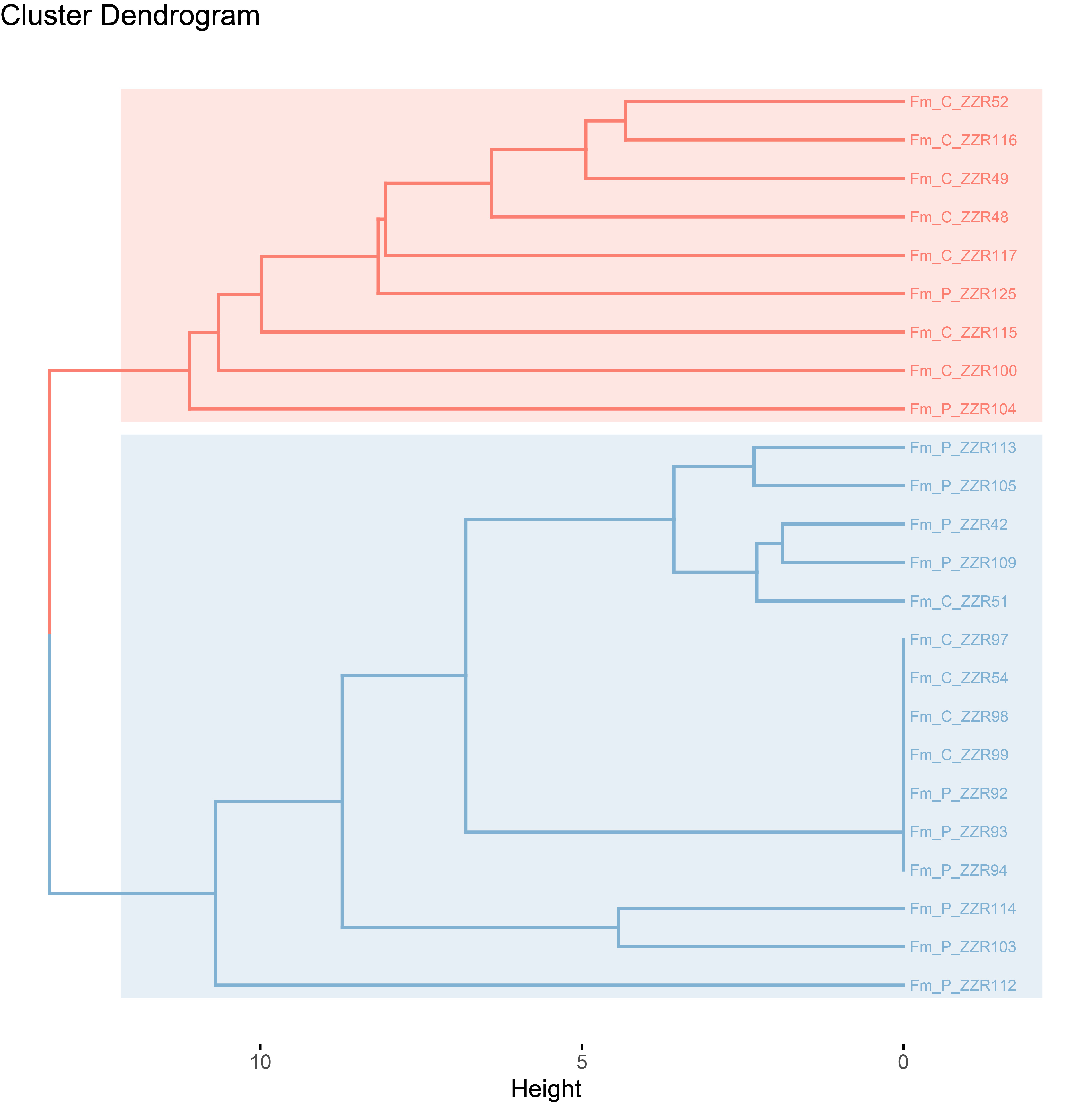

Supplement: SUPPLEMENTARY FIGURE S3 — The cluster dendrogram produced by the ward.D2 method using Euclidean distances based on the compositions of Wolbachia bacteria in samples. The tip labels in the dendrogram are the sample codes; Fm_C represents the cheater of F. microcarpa; Fm_P represents the pollinator of F. microcarpa. [file Image_3.JPEG]

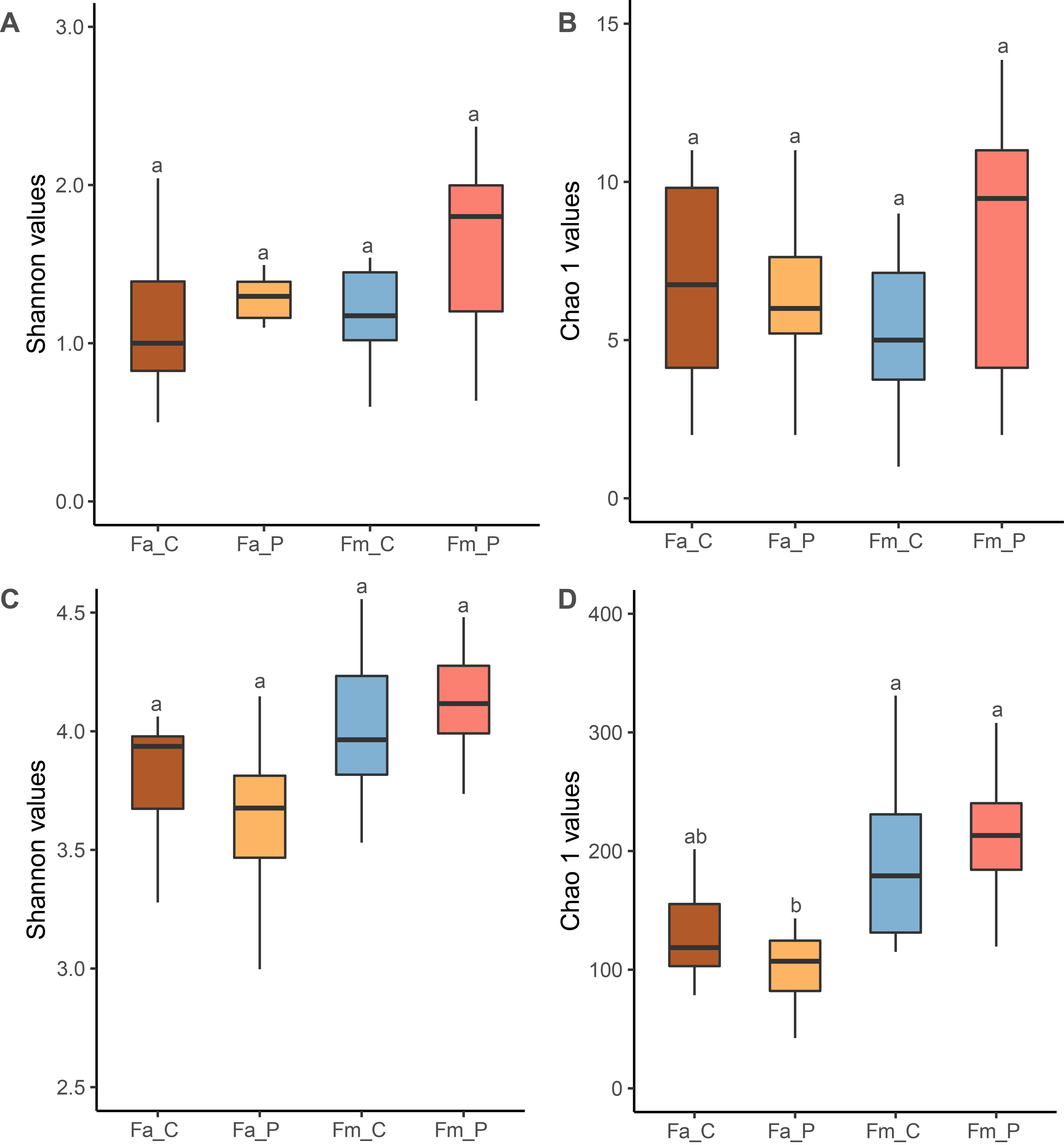

Supplement: SUPPLEMENTARY FIGURE S4 — The diversity indices patterns of fig wasps-associated fungi and bacteria. (A,B) The Shannon and Chao 1 diversity index of fungal communities. (C,D) The Shannon and Chao 1 diversity index of bacterial communities. The letters at the top of each panel were calculated by the least significant difference (LSD) test, the same letter represents no significant difference. Fa_C represents the cheater of F. altissima; Fa_P represents the pollinator of F. altissima; Fm_C represents the cheater of F. microcarpa; Fm_P represents the pollinator of F. microcarpa. [file Image_4.JPEG]

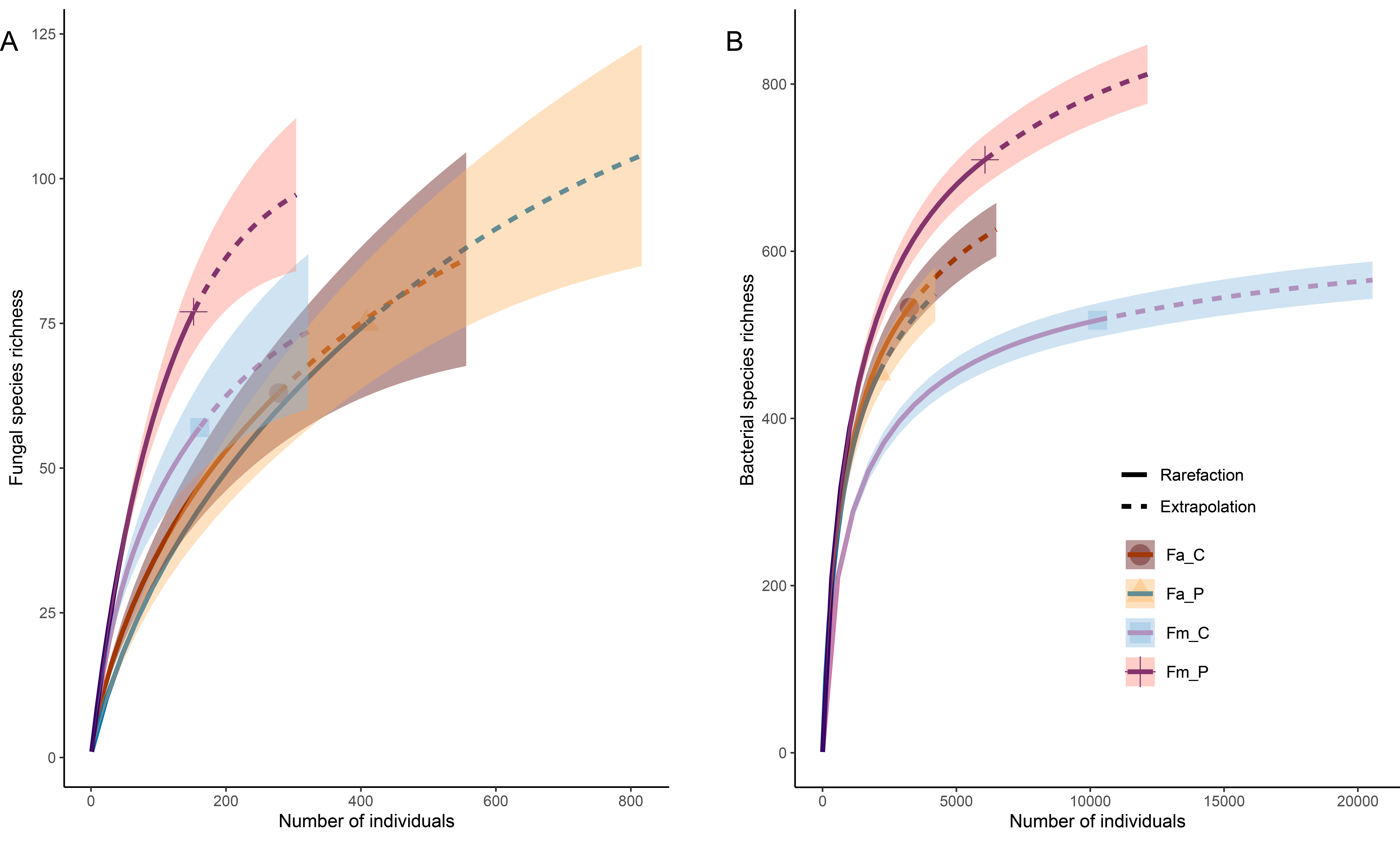

Supplement: SUPPLEMENTARY FIGURE S6 — The rarefaction curves of fungal community (A) and bacterial (B) among four different samples. Fa_C represents the cheater of F. altissima; Fa_P represents the pollinator of F. altissima; Fm_C represents the cheater of F. microcarpa; Fm_P represents the pollinator of F. microcarpa. [file Image_6.JPEG]

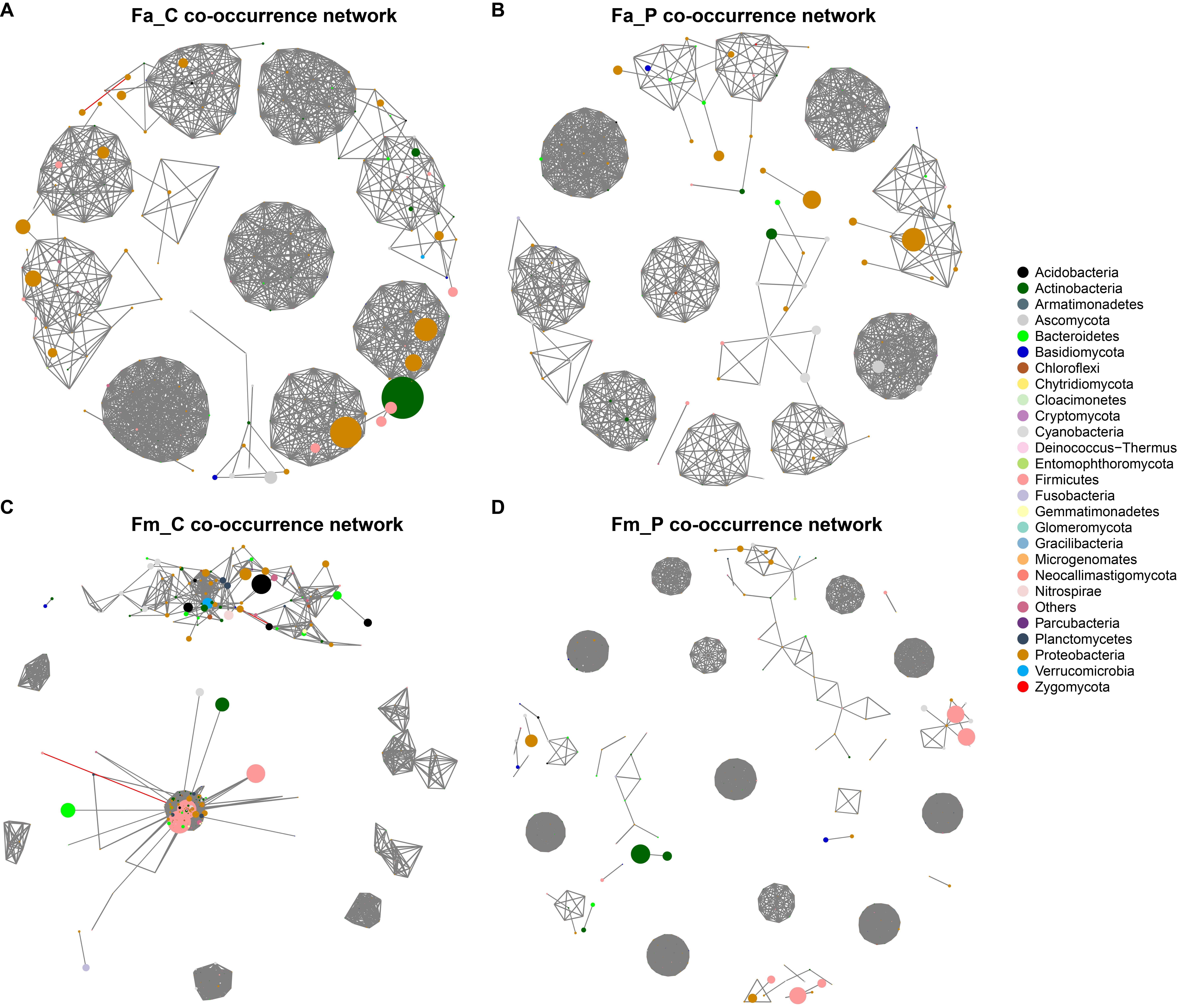

Supplement: SUPPLEMENTARY FIGURE S7 — The microbial co-occurrence networks based on filtered correlation coefficient (Spearman’s r > 0.8 and p value <0.05). (A) The co-occurrence network from the cheater of F. altissima. (B) The co-occurrence network from the pollinator of F. altissima. (C) The co-occurrence network from the cheater of F. microcarpa. (D) The co-occurrence network from the pollinator of F. microcarpa. The nodes in the co-occurrence network are the microbial genera, the color of nodes represents the phylum that the genus belongs to, the links in the network are the significant correlation in two nodes, the width of link line reflects the absolute value of correlation coefficient and the color of link line represents the positive (gray) and negative (red) correlation coefficient. Fa_C represents the cheater of F. altissima; Fa_P represents the pollinator of F. altissima; Fm_C represents the cheater of F. microcarpa; Fm_P represents the pollinator of F. microcarpa. [file Image_7.JPEG]

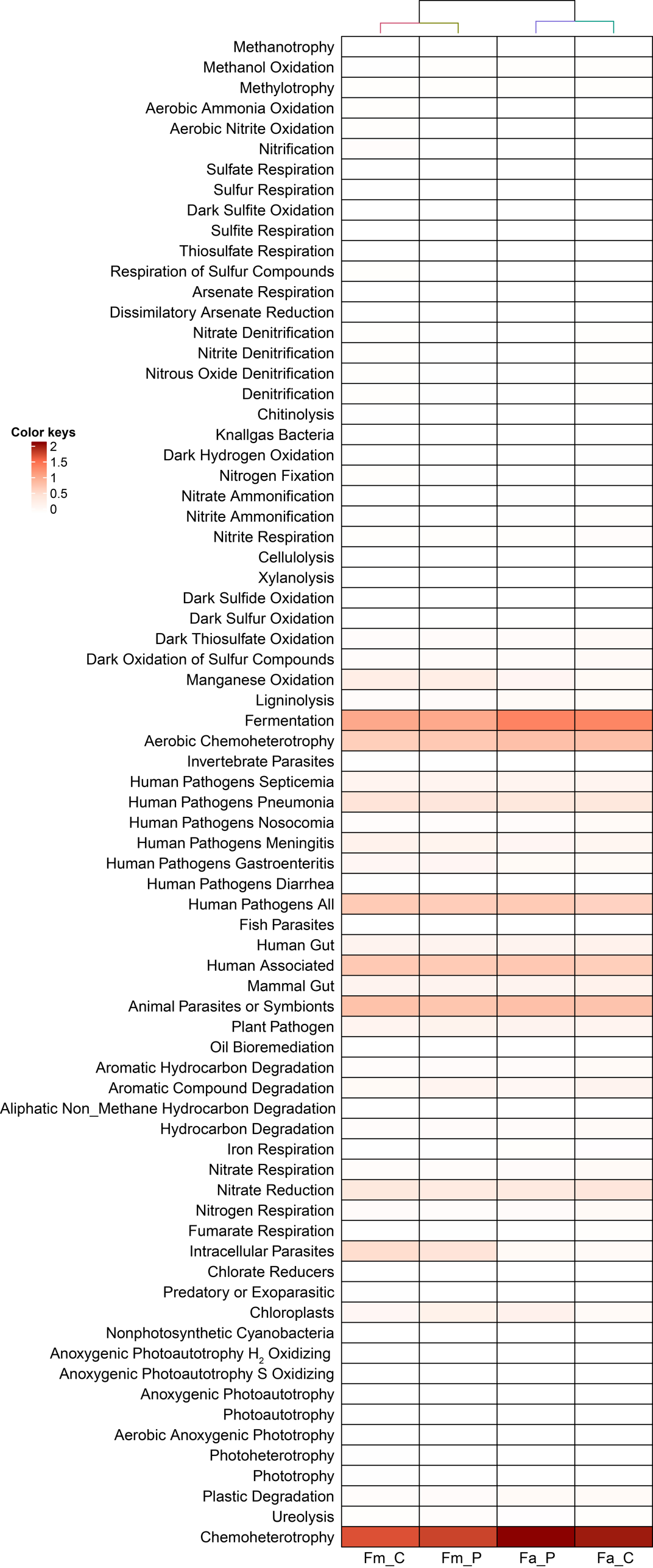

Supplement: SUPPLEMENTARY FIGURE S8 — Heatmap of the functional groups obtained from FAPROTAX for each sample of bacterial community. The color bar reflects the value of functional abundance. Fa_C represents the cheater of F. altissima; Fa_P represents the pollinator of F. altissima; Fm_C represents the cheater of F. microcarpa; Fm_P represents the pollinator of F. microcarpa. [file Image_8.JPEG]
