# Supplementary material for: Diversity and metabolic potentials of microbial communities associated with pollinator and cheater fig wasps in fig-fig wasp mutualism system
Source: Front Microbiol. 2022 Nov 18;13:1009919. doi: 10.3389/fmicb.2022.1009919 (PMC9715610; doi:10.3389/fmicb.2022.1009919)
Supplement: Supplementary file 9 [file Data_Sheet_1.PDF]

Table S1 The sequencing quality information of samples.

Sample	Species	Longitude	Latitude	Raw Data (reads)	Q20 (%)	GC Content (%)	Clean reads after trim	The genome coverage (Mb, >=5*	Mean depth (>=5*)
K.gibbosae_349	*Kradibia gibbosae*	101.26062	21.931362	31,684,796	97.72 (95.75)	35.19 (35.40)	31,407,306	123.32	18.26
Fa_C_ZZR34	*F. altissima* cheater	101.26062	21.931362	26,245,714	97.95 (97.61)	29.58 (29.70)	25,582,310	154.57	19.14
Fa_C_ZZR35	*F. altissima* cheater	101.26062	21.931362	46,461,553	98.14 (97.14)	29.66 (29.71)	45,555,312	186.64	29.10
Fa_C_ZZR36	*F. altissima* cheater	101.26062	21.931362	22,955,303	98.04 (96.80)	29.91 (30.03)	22,512,076	148.61	17.22
Fa_C_ZZR37	*F. altissima* cheater	101.26062	21.931362	22,289,038	97.80 (97.42)	29.23 (29.37)	21,624,095	145.52	17.16
Fa_C_ZZR38	*F. altissima* cheater	101.26062	21.931362	39,733,351	97.94 (96.88)	29.86 (30.03)	39,090,752	178.88	25.59
Fa_C_ZZR39	*F. altissima* cheater	101.26062	21.931362	42,607,573	98.22 (97.07)	29.82 (29.85)	41,936,487	182.20	27.35
Fa_C_ZZR4	*F. altissima* cheater	98.853128	25.851588	23,416,929	97.82 (96.87)	29.96 (30.06)	23,080,402	149.21	17.82
Fa_C_ZZR80	*F. altissima* cheater	98.853128	25.851588	24,399,015	97.58 (96.65)	31.32 (31.35)	24,092,184	149.11	17.74
Fa_C_ZZR81	*F. altissima* cheater	98.853128	25.851588	27,641,730	97.38 (96.08)	31.10 (31.25)	27,234,959	157.29	19.79
Fa_C_ZZR82	*F. altissima* cheater	98.853128	25.851588	34,274,827	97.23 (96.22)	31.70 (31.85)	33,899,440	169.71	23.17
Fa_C_ZZR83	*F. altissima* cheater	98.853128	25.851588	25,450,793	97.67 (96.62)	31.71 (31.71)	25,136,239	152.66	18.81
Fa_C_ZZR86	*F. altissima* cheater	98.853128	25.851588	23,761,147	97.63 (97.47)	30.57 (30.63)	23,271,845	148.69	17.85
Fa_P_ZZR2	*F. altissima* pollinator	101.26062	21.931362	23,946,858	98.08 (96.88)	32.27 (32.40)	23,561,566	148.38	17.34
Fa_P_ZZR3	*F. altissima* pollinator	101.26062	21.931362	24,872,385	98.19 (97.19)	31.21 (31.29)	24,408,960	152.52	17.69
Fa_P_ZZR31	*F. altissima* pollinator	101.26062	21.931362	19,621,552	98.08 (96.88)	31.75 (31.89)	19,232,169	136.82	14.90
Fa_P_ZZR32	*F. altissima* pollinator	101.26062	21.931362	39,916,065	97.93 (96.03)	30.54 (30.91)	39,297,537	180.00	24.46
Fa_P_ZZR33	*F. altissima* pollinator	101.26062	21.931362	22,536,741	98.13 (97.03)	31.14 (31.22)	22,041,931	47.37	16.17
Fa_P_ZZR5	*F. altissima* pollinator	99.178387	25.111944	31,267,467	97.87 (95.92)	30.27 (30.34)	30,644,167	166.64	20.58
Fa_P_ZZR6	*F. altissima* pollinator	99.178387	25.111944	28,560,798	97.30 (96.68)	30.23 (30.29)	28,001,480	161.03	19.46
Fa_P_ZZR78	*F. altissima* pollinator	98.853128	25.851588	22,404,747	97.48 (96.44)	31.19 (31.32)	22,196,475	146.70	16.31
Fa_P_ZZR79	*F. altissima* pollinator	98.853128	25.851588	36,854,660	97.63 (96.60)	31.39 (31.43)	36,455,635	175.21	23.18
Fa_P_ZZR84	*F. altissima* pollinator	98.853128	25.851588	21,845,299	97.57 (96.34)	31.57 (31.63)	21,605,466	143.45	15.39
Fa_P_ZZR85	*F. altissima* pollinator	98.853128	25.851588	27,879,969	97.65 (96.49)	31.04 (31.08)	27,437,865	160.10	19.01
Fa_P_ZZR87	*F. altissima* pollinator	98.853128	25.851588	33,654,893	97.74 (96.18)	30.70 (30.69)	33,269,125	170.85	21.85
Fm_C_ZZR100	*F.microcarpa* cheater	98.808048	25.294557	20,488,968	97.41 (96.42)	33.12 (33.24)	20,270,541	307.62	15.93
Fm_C_ZZR115	*F.microcarpa* cheater	101.26062	21.931362	21,291,998	96.77 (95.99)	32.61 (32.81)	21,097,868	306.05	16.78
Fm_C_ZZR116	*F.microcarpa* cheater	101.26062	21.931362	23,310,387	96.40 (94.73)	33.39 (33.70)	23,113,232	304.89	18.54
Fm_C_ZZR117	*F.microcarpa* cheater	101.26062	21.931362	23,909,722	96.87 (95.91)	32.57 (32.73)	23,648,172	316.56	18.35
Fm_C_ZZR48	*F.microcarpa* cheater	101.26062	21.931362	39,249,659	98.20 (96.69)	31.28 (31.36)	38,600,984	331.26	29.06
Fm_C_ZZR49	*F.microcarpa* cheater	101.26062	21.931362	28,582,574	97.92 (95.25)	31.25 (31.35)	28,085,322	327.16	21.46
Fm_C_ZZR51	*F.microcarpa* cheater	101.26062	21.931362	21,758,798	97.31 (96.53)	31.30 (31.42)	21,365,320	320.69	16.21
Fm_C_ZZR52	*F.microcarpa* cheater	101.26062	21.931362	23,424,376	97.73 (97.20)	31.34 (31.41)	22,909,622	322.81	17.67
Fm_C_ZZR54	*F.microcarpa* cheater	101.26062	21.931362	25,860,632	97.21 (96.46)	30.94 (31.05)	25,239,129	324.29	19.37
Fm_C_ZZR97	*F.microcarpa* cheater	98.808048	25.294557	42,403,026	97.04 (94.99)	33.24 (33.52)	42,075,367	328.94	31.32
Fm_C_ZZR98	*F.microcarpa* cheater	98.808048	25.294557	24,799,000	98.02 (96.80)	32.21 (32.37)	24,589,568	320.79	18.84
Fm_C_ZZR99	*F.microcarpa* cheater	98.808048	25.294557	23,828,931	98.09 (97.01)	33.34 (33.47)	23,588,863	307.96	18.75
Fm_P_ZZR103	*F.microcarpa* pollinator	98.808048	25.294557	23,779,928	98.31 (97.25)	32.84 (32.91)	23,535,155	360.20	19.07
Fm_P_ZZR104	*F.microcarpa* pollinator	102.865909	23.934295	24,784,292	98.16 (96.74)	32.94 (33.02)	24,547,052	362.22	19.70
Fm_P_ZZR105	*F.microcarpa* pollinator	102.865909	23.934295	24,973,088	98.30 (97.12)	32.61 (32.69)	24,773,158	363.83	19.52
Fm_P_ZZR109	*F.microcarpa* pollinator	101.518069	25.055846	25,256,165	98.13 (96.96)	32.25 (32.38)	25,064,729	365.96	19.94
Fm_P_ZZR112	*F.microcarpa* pollinator	101.518069	25.055846	23,731,701	98.28 (97.33)	32.30 (32.37)	23,541,685	371.10	18.61
Fm_P_ZZR113	*F.microcarpa* pollinator	101.518069	25.055846	23,668,013	98.02 (96.70)	32.54 (32.71)	23,483,449	366.42	18.80
Fm_P_ZZR114	*F.microcarpa* pollinator	101.518069	25.055846	22,180,348	96.96 (96.23)	32.36 (32.50)	21,988,664	364.14	17.55
Fm_P_ZZR125	*F.microcarpa* pollinator	101.564056	21.462321	26,833,013	97.84 (96.32)	33.57 (33.68)	26,585,623	357.23	19.37
Fm_P_ZZR42	*F.microcarpa* pollinator	101.26062	21.931362	30,593,537	97.77 (95.26)	30.83 (30.95)	29,839,539	376.32	23.16
Fm_P_ZZR92	*F.microcarpa* pollinator	98.808048	25.294557	20,637,368	97.34 (96.53)	32.05 (32.19)	20,425,455	364.92	16.19
Fm_P_ZZR93	*F.microcarpa* pollinator	98.808048	25.294557	22,142,920	97.48 (96.90)	31.86 (31.93)	21,940,354	370.21	17.33
Fm_P_ZZR94	*F.microcarpa* pollinator	98.808048	25.294557	24,692,979	98.14 (97.12)	32.40 (32.53)	24,489,058	367.36	19.48
